# Web-Based Medical Information Searching by Chinese Patients With Breast Cancer and its Influence on Survival: Observational Study

**DOI:** 10.2196/16768

**Published:** 2020-04-17

**Authors:** Yan Li, Shan Ye, Yidong Zhou, Feng Mao, Hailing Guo, Yan Lin, Xiaohui Zhang, Songjie Shen, Na Shi, Xiaojie Wang, Qiang Sun

**Affiliations:** 1 Department of Breast Surgery Peking Union Medical College Hospital Peking Union Medical College, Chinese Academy of Medical Sciences Beijing China

**Keywords:** breast cancer, internet, disease-free survival, breast conserving therapy, online information, satisfaction level

## Abstract

**Background:**

The internet allows patients to easily look for health information. However, how Chinese patients with breast cancer use the internet has rarely been investigated, and there is a scarcity of information about the influence of internet use on survival.

**Objective:**

This observational study aimed to investigate the details of online medical information searching by Chinese patients with breast cancer and to determine whether internet use has any survival benefits.

**Methods:**

Patients who were diagnosed with invasive breast cancer at Peking Union Medical College Hospital between January 2014 and December 2015 were enrolled. We obtained information on their internet-searching behavior and gathered data from the patients’ medical and follow-up records. The associations between internet use and other clinic-pathological factors were analyzed. A Cox proportional-hazards model and the Kaplan-Meier method were used for disease-free survival (DFS) analyses.

**Results:**

A total of 973 patients with invasive breast cancer who underwent definitive surgery took part in the study. Among them, 477 cases (49.0%) performed web-based breast cancer information searching before the initial treatment. A multivariate logistic regression analysis suggested that web-based breast cancer information searching was significantly associated with younger age (odds ratio [OR] 0.95, 95% CI 0.94-0.97, *P*<.001), higher education level (OR 1.37, 95% CI 1.01–1.86, *P*=.04), and breast conserving surgery (OR 1.35, 95% CI 1.04-1.77, *P*=.03). Baidu (73.4%, 350/477) and WeChat (66.7%, 318/477) were the two most popular online information sources for breast cancer; however, only 44.9% (214/477) felt satisfied with the online information. In contrast to the nonweb searching group, the web-using patients who were satisfied with online information showed significantly improved DFS (hazard ratio 0.26; 95% CI 0.08-0.88, *P*=.03).

**Conclusions:**

The patients who were most likely to search the internet for breast cancer information were younger and well-educated, and they were more likely to have breast conserving therapy. Web-using patients who were satisfied with the internet information showed significantly improved DFS. Patients should browse credible websites offering accurate and updated information, and website developers should provide high-quality and easy-to-understand information to better meet the needs of patients with breast cancer.

## Introduction

The general population has been increasing their use of the internet since the early 1990s. By the end of 2017, China had 772 million internet users, which is the largest population of “netizens” in the world. The internet penetration rate reached 55.8%, with increases of 2.6% per year, which was higher than the world average. The number of mobile internet users in China increased to 753 million by the end of 2017, and there was a yearly increase of 57.34 million [1].

The internet enables patients to conveniently search for health information and it now plays a larger part in their treatment decision making [2]. Using health information from the internet is associated with stronger participation in decision making, better decisions, and more frequent changes in health behavior, and it may enable patients to communicate with doctors more effectively [2,3]. Some surveys have found considerably high rates of online health information-searching behavior between patients who are more educated, younger, and of a higher socioeconomic status [4,5]. However, other studies showed that educational level and residence had no effect on the rates of patients searching the internet for health information [4,6]. Although searching for internet medical information may benefit patients’ understanding of the disease and medical processes, some studies showed there is a significant lack of high-quality websites providing accurate and complete information [7-9]. Many websites provide inaccurate, conflicting, or misleading information on guidelines and recommendations established by professional medical groups, and it may lead to confusion, unnecessary anxiety, or treatment delay [10,11].

Many studies have revealed a significant proportion of patients with cancer use the internet to find information about their diseases [12,13]. To date, most of the studies about online cancer information research are carried out in Europe and North America [14,15]. Castleton reported that about 63% of North American patients with cancer searched the internet for health-related information [16]. Breast cancer is the most frequent cancer that occurs in Chinese women, and there is an age-standardized rate of 21.6 cases per 100,000 women [17]. For patients with breast cancer in western countries, the rate of internet use for health-related information was reported to be between 29% and 57% [12,18,19]. Patients with breast cancer search the internet for information related to treatment, effect on daily life, prognosis, and cause of disease [18,19]. Internet information is also important in patients’ decision making regarding the type of surgery for breast cancer [20]. However, the use of the internet has rarely been investigated in Chinese patients with breast cancer, and there is a scarcity of information about the influence of internet use on the recurrence and survival of breast cancer.

In this observational study, we examined how patients with breast cancer use the internet to obtain information pertaining to their disease. The aims of our study were to identify the factors that relate to the use of the internet as a source of health information, investigate the details of online information searching, and determine whether internet use would have any survival benefits for patients with breast cancer. We hope that by recognizing the online information searching details of patients, we will be able to suggest strategies to facilitate internet searches on breast cancer information and help patients better cope with their disease.

## Methods

### Ethics Statement

This study was endorsed by the ethics committee of Peking Union Medical College Hospital (PUMCH), and all the participants provided informed consent.

### Patients

Patients who were diagnosed with invasive breast cancers in PUMCH between January 2014 and December 2015 were enrolled in this study. To be included, the participants must have had definitive surgery of both the breast and axilla. The exclusion criteria were as follows: neoadjuvant therapy received prior to definitive surgery or distant metastasis of breast cancer (Stage IV). All eligible participants had pertinent medical records, and they had regular follow-ups. The last follow-up date was January 8, 2019.

### Data Collection

On the day of discharge from the hospital, the patients, who had just received a definitive surgery of breast cancer, were approached by one member of the research team. The researcher explained the process and significance of the study. After the patients signed the statement of informed consent, the researcher had a face-to-face interview with them. The interview aimed to investigate the web-based breast cancer information searching from the detection of a breast tumor to the initial treatment. The survey included 7 questions relating to sociodemographics (age, education, and residence), web-based information-seeking information (devices, channels and websites, and satisfaction level with web-based information), and self-rated anxiety level.

We gathered data on tumor characteristics from the patients’ medical records, such as molecular subtype, cancer stage, and type of surgery. Follow-up data were retrieved from the database. The patients treated at PUMCH were instructed to return for follow-up visits every 6 months after the surgery. A follow-up via telephone was used if a patient failed to attend their appointment. The follow-up procedures included medical history, physical examination, laboratory tests, and imaging examinations (eg, ultrasound, mammography, computed tomography scan, or bone scan).

The endpoint of this study was disease-free survival (DFS). DFS was calculated from the initial treatment to a second primary cancer, recurrence, or death without evidence of recurrence or second primary cancer. Breast cancer recurrence included distant metastases and locoregional recurrence.

### Statistical Analysis

Quantitative data were compared with a Student’s *t* test. Categorical data were compared with a 2-sided chi-square test and multivariate logistic regression model. DFS was studied using the Kaplan-Meier curve. Time-to-event endpoints were analyzed with 2-sided log-rank tests. A multivariate Cox proportional-hazards regression model survival analysis was performed after adjusting potential factors that might affect the patient’s survival, including age, molecular subtype, stage, and type of surgery. Differences were considered significant at *P*<.05. Statistical analyses were performed using the STATA statistical software package (version 14.0).

### Data Availability Statement

All the data that were generated or analyzed in this study are included in this article.

## Results

### Patient

A total of 1004 patients with invasive breast cancer who underwent definitive surgery were eligible for this study and 31 of them refused. All 973 final participants were female and of Chinese descent. The median age was 47.2 years (range 21-79); 548 (56.3%) of the patients had breast conserving surgery, and 425 (43.7%) of them had a mastectomy. Postoperative adjuvant treatment was determined according to the National Comprehensive Cancer Network guidelines and the patient’s actual situation.

### Web Use

Among the 973 participants, 477 cases (49.02%) performed web-based breast cancer information searching before initial treatment (web group), while 496 cases (50.98%) did not seek medical information via the internet (nonweb group). By *t* test, age was significantly younger in the web group. Analyses with a chi-square test showed that tumor stage, molecular subtype, and self-rated anxiety levels were not significantly different between the two groups. However, higher education level, urban residence, and breast conserving therapy (BCT) were significantly associated with the web group (Table 1).

**Table 1:** Patient characteristics within subgroups.

Characteristic		Web group (n=477)	Nonweb group (n=496)	*P* value
Age (years), mean (SD)		43.81 (11.73)	50.49 (14.00)	<.001
**Education, n (%)**				.01
	< high school	376 (78.8)	356 (71.8)	
	< high school	101 (21.2)	140 (28.2)	
**Residence, n (%)**				<.001
	Urban	278 (58.3)	211 (42.5)	
	Rural	199 (41.7)	285 (57.5)	
**Stage, n (%)**				.14
	I	244 (51.2)	224 (45.2)	
	II	141 (29.6)	157 (31.7)	
	III	92 (19.3)	115 (23.2)	
**Molecular subtype, n (%)**				.79
	Luminal A	223 (46.8)	246 (49.6)	
	Luminal B	112 (23.5)	113 (22.8)	
	Triple-negative breast cancer	95 (19.9)	95 (19.2)	
	HER2^a^-positive	47 (9.9)	42 (8.5)	
**Self-rated anxiety level, n (%)**				.52
	Low	225 (47.2)	251 (50.6)	
	Medium	125 (26.2)	126 (25.4)	
	High	127 (26.6)	119 (24.0)	
**Type of surgery, n (%)**				.047
	Breast conserving therapy	284 (59.5)	264 (53.2)	
	Mastectomy	193 (40.5)	232 (46.8)	

^a^HER2: human epidermal growth factor receptor 2.

A multivariate logistic regression was also carried out. The outcomes suggested that web-based breast cancer information searching was significantly associated with younger age, higher education, and breast conserving surgery. However, for residence, tumor stage, molecular subtype, and self-rated anxiety level, correlations with web-based information searching were not significant (Table 2).

**Table 2:** Multivariate logistic analysis of the association between web-based information seeking and patient characteristics.

Characteristic		Web use (n=477), n (%)	Odds ratio (95% CI)	*P* value
Age		N/A^a^	0.95 (0.94-0.97)	<.001
**Education**				
	< high school (reference)	101 (41.9)	N/A	N/A
	< high school	376 (51.4)	1.37 (1.01-1.86)	.04
**Residence**				
	Rural (reference)	199 (41.1)	N/A	N/A
	Urban	278 (56.9)	0.78 (0.53-1.14)	.197
**Stage**				
	I (reference)	244 (52.1)	N/A	N/A
	II	141 (47.3)	0.81 (0.59-1.09)	.16
	III	92 (44.4)	0.73 (0.53-1.03)	.08
**Molecular subtype**				
	Luminal A (reference)	223 (47.6)	N/A	N/A
	Luminal B	112 (49.8)	1.01 (0.71-1.41)	.97
	Triple-negative breast cancer	95 (50.0)	1.04 (0.73-1.48)	.83
	HER2^b^-positive	47 (52.8)	1.20 (0.75-1.93)	.45
**Self-rated anxiety level**				
	Low (reference)	225 (47.3)	N/A	N/A
	Medium	125 (49.8)	1.03 (0.75-1.42)	.86
	High	127 (51.6)	1.13 (0.82-1.56)	.45
**Type of surgery**				
	Mastectomy (reference)	193 (45.4)	N/A	N/A
	Breast conserving therapy	284 (51.8)	1.35 (1.04-1.77)	.03

^a^N/A: not applicable.

^b^HER2: human epidermal growth factor receptor 2.

We further investigated the details of web-based breast cancer information-seeking behavior, which are shown in Table 3. Regarding web channels, Baidu (Chinese “Google”) and WeChat (Chinese “Facebook”) were the top two most popular online information sources for breast cancer. Less than half of participants browsed professional websites.

**Table 3:** Details of patients’ web-based breast cancer information-searching behavior.

Variables		Web-using participants (n=477), n (%)
**Devices**		
	Computer (desktop, laptop, tablets)	178 (37.3)
	Mobile phones	203 (42.6)
	Both	96 (20.1)
**Web channels**		
	Baidu	350 (73.4)
	WeChat	318 (66.7)
	Professional websites	213 (44.7)
	Discussion forums	129 (27.0)
	Others	147 (30.8)
**Satisfaction level**		
	Satisfied	214 (44.9)
	Unsatisfied	263 (55.1)

### Survival Analysis Between the Web Group and Nonweb Group

**Figure 1:**
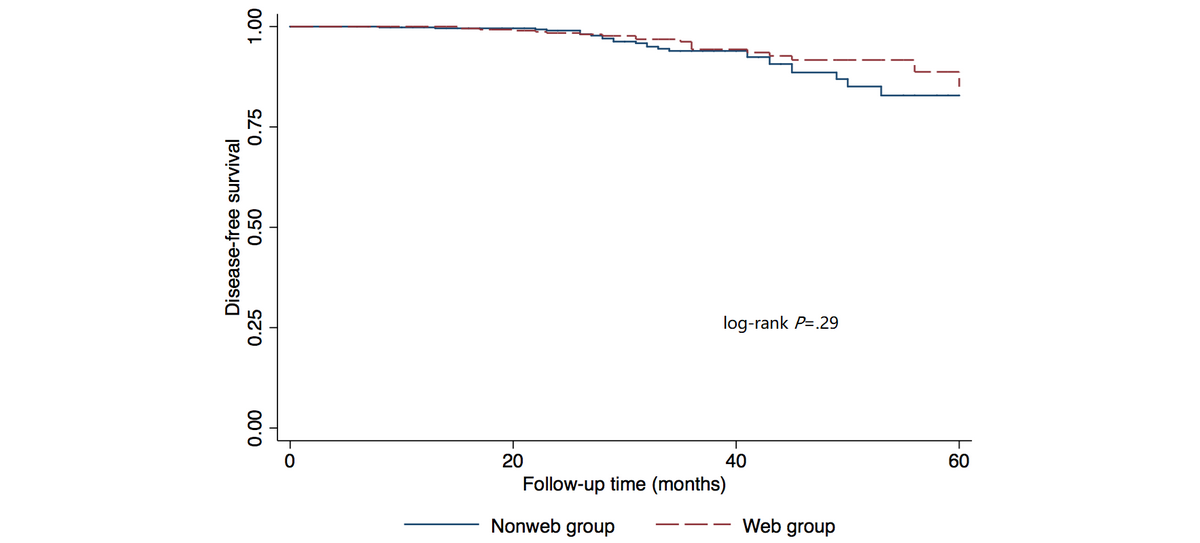
Kaplan-Meier survival curves of disease-free survival by web usage.

**Figure 2:**
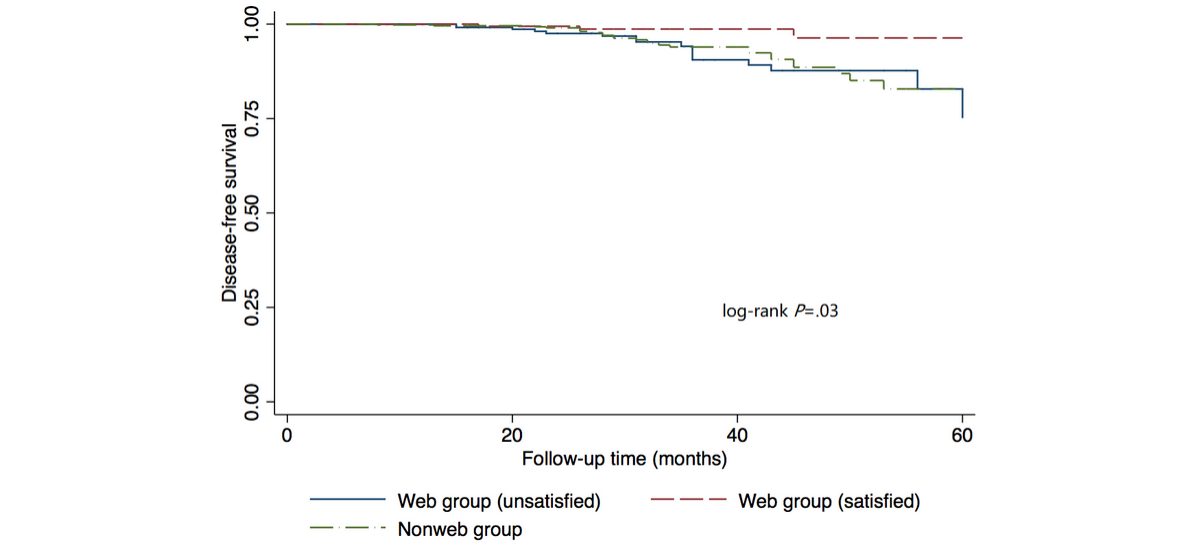
Kaplan-Meier survival curves of disease-free survival (DFS) according to web usage and satisfaction level of internet information.

The median follow-up time was 35 months (range 12-60). In the web group, there were 19 DFS events: 10 cases of bone metastases, 3 cases of locoregional recurrence, 3 cases of lung metastases, 1 case of distant lymph node metastasis, 1 case of liver metastasis, and 1 case of multiple metastases. In the nonweb group, there were 26 DFS events: 11 cases of bone metastases, 6 cases of locoregional recurrence, 2 cases of lung metastases, 2 cases of distant lymph node metastases, 2 cases of liver metastases, 1 case of brain metastasis, 1 case of multiple metastases, and 1 case of death. The 3-year Kaplan-Meier estimates for DFS were 94.32% in the web group, and 93.94% in the nonweb group (Figure 1). The log-rank comparison showed no significant difference in DFS (*P*=.29). If we further divided the web group into a “Satisfied” subgroup and an “Unsatisfied” subgroup, the log-rank comparison showed a significant difference among all three groups (web-satisfied, web-unsatisfied, and nonweb) (*P*=.03; Figure 2).

### Multivariate Analysis for DFS

In the multivariate analysis, the included prognostic factors for DFS were age, tumor stage, molecular subtype, type of surgery, and web-seeking condition (Table 4). Compared to the nonweb group, the web-using patients who were unsatisfied with the internet information showed no significant difference for DFS; however, the web-using patients who were satisfied with the internet information showed significantly improved DFS, after adjustment for all other factors. The web-satisfied subgroup also showed significantly improved DFS than the web-unsatisfied subgroup (hazard ratio 0.26; 95% CI, 0.08-0.90, *P*=.03).

**Table 4:** Cox proportional hazards regression model analysis of disease-free survival.

Factor		Hazard ratio (95% CI)	*P* value
Age (continuous)		1.00 (0.98-1.03)	.54
**Stage**			
	I (reference)	N/A^a^	N/A
	II	1.20 (0.57-2.51)	.63
	III	1.86 (0.90-3.82)	.09
**Molecular subtype**			
	Luminal A (reference)	N/A	N/A
	Luminal B	2.12 (0.86-5.23)	.10
	Triple-negative breast cancer	3.79 (1.63-8.77)	.002
	HER2^b^-positive	7.11 (2.89-17.48)	<.001
**Type of surgery**			
	Mastectomy (reference)	N/A	N/A
	Breast conserving therapy	1.85 (0.98-3.48)	.06
**Web-based information seeking condition**			
	No web (reference)	N/A	N/A
	Web use-unsatisfied	0.99 (0.51-1.96)	.99
	Web use-satisfied	0.26 (0.08-0.88)	.03

^a^N/A: not applicable.

^b^HER2: human epidermal growth factor receptor 2.

## Discussion

The use of the internet for obtaining health information has expanded significantly in the past several years. Because of its frequently updated content, wide availability, and multimedia forms of presenting data, many patients use the internet to acquire health information [21-23]. The reported rates of internet use by patients with cancer have varied widely, with estimates ranging from 4.8% to 77% [19,24-28]. The rate of 49.0% (477/973) in this study is within this range, and it is consistent with the data available specifically for breast cancer: 21% to 51% [12,19,24].

The results from this study showed a definite association between education level, age, location of residence, BCT, and the likelihood that a patient uses the internet for breast cancer information. However, tumor stage, molecular subtype, and anxiety were not as statistically significant as factors affecting internet use among patients with breast cancer. Multivariate analysis suggested that age, education level, and type of surgery were associated with use of the internet but not location of residence. The current literature has reported similar findings [19,24,25]. Younger patients have more opportunities to access the internet, and they are more open to online information, so the link between age and internet use is easily understood [1]. For older adults, there are usually more obstacles to access the internet. They are less familiar with the necessary skills to browse the webpages (eg, typing or setting up an account) and the basic logic of doing an online search. Assistance from family, community, and government is needed to help them overcome these difficulties. Some older adults may also have physical problems that limit their use of the internet, such as low vision and trembling hands. Special software such as interactive voice response systems need to be developed and provided to them [15,22]. As for education level, a possible explanation might be that people with lower education are more likely to have lower socioeconomic status and a lower income level. This has led to concerns about a “digital divide”, that is, the existence of a barrier for patients in lower educational and socioeconomic groups to benefit from online health information [29]. To eliminate or reduce this “digital divide”, training programs of internet use should be offered to these less educated people. In addition, the government should build high-speed and more affordable broadband infrastructure and offer subsidies to those who cannot afford the cost of basic internet devices [29-31]. However, in our multivariate analysis, the likelihood of internet use was not different between urban and rural residences, which is not consistent with previous studies [30,31]. In China, because of the lack of an effective appointment and referral system, receiving medical service in a large hospital in a metropolis such as Beijing and Shanghai is an expensive, complicated, and time-consuming process [32]. Founded in 1921 by the Rockefeller Foundation, PUMCH is a national center guiding the diagnosis and treatment of difficult and serious diseases appointed by the National Health Commission, and it ranks first in a series of different ranking systems [33]. As a result, the rural residents who can visit PUMCH are more likely to have a relatively higher economic status compared to those living in remote and poor areas. For these people, the internet using condition is likely to be similar to that of urban residents. Further studies should pay more attention to those people with extreme poverty who are in more need of various sources of medical information.

Our study implied that patients with breast cancer who were searching for online medical information were more likely to receive BCT. Jordan et al [34] reported that high-quality and easily accessible web-based information could help to significantly improve patients’ participation in decision making for breast cancer surgery. Currently in China, the rates of BCT are relatively low compared with the international average level [35]. One of the reasons is that the patients lack knowledge about BCT, which is misunderstood to be “unsafe” or “incomplete”, and so they may opt to avoid this treatment even when it is suggested by their clinician [35]. The internet can play an important role in educating patients with breast cancer to accept less invasive treatments as the standard procedure if they are indicated as being the most suitable treatment by their clinicians [36].

We also reported that the self-rated anxiety level was not significantly different whether the patients sought online medical information or not. On the one hand, high-quality online information can facilitate patients’ understanding of the disease, and make them more prepared to face it, both physically and psychologically [37]. On the other hand, the online information is usually too complex and misleading for the general public to comprehend, and it sometimes even contains obvious mistakes. This will lead to confusion and anxiety, and offset the possible improvement of psychological well-being brought by internet searching [34,38,39]. However, some studies reported that online communities, forums, and peer-support groups, which offer the patients more opportunities to express their feelings and receive peer education, have the potential to improve the emotional well-being of patients with cancer [40-42]. Therefore, online interactive and individualized social media tools about breast cancer should be developed to enable patients to establish one-to-one, one-to-many, or many-to-many communications with professionals and their peers [43].

This study demonstrated that, altogether, more than 62% of the web group used mobile smartphones to search for online information on breast cancer. By the end of 2017, the percentage of Chinese netizens using mobile phones to access the internet was 97.5%, hitting a new high record [1]. This was 2.4 points more than at the end of 2016. WeChat is an innovative application that is installed on over 90% of smartphones and 60% of computers, and it is currently performing as an important tool that is transforming the daily life of users in various ways [1]. Many different types of medical information were continuously created and transmitted among millions of users through WeChat, which provided WeChat with the enormous potential to affect the general public’s health knowledge and possible health status. In our study, 66.7% (318/477) of the web group searched for breast cancer information via WeChat. However, Baidu is still on the top of the web channel list, with 73.4% (350/477) of the web group using Baidu as the information source. The largest concern connected with the internet as a health resource is that it is unregulated; high-quality information sits side-by-side with poor or misleading information. In this study, the general satisfaction level with internet information was not high, with a satisfaction rate of only 44.9% (214/477). This rate is consistent with previous articles [18]. Accuracy and readability are the most concerning issues about online medical information. Patients should be recommended web channels with professional sources, updated information, interactive communication, and patient-friendly language [8,13,14].

Regarding survival analysis, univariate and multivariate regression analyses indicated that there was no DFS difference between the web and nonweb groups; however, web-using patients who were satisfied with internet information showed significantly improved DFS. This result can be explained as follows: accurate online information is beneficial to patients’ recurrence and survival condition, while inaccurate or “harmful” information can offset this positive effect. Misled by harmful information, patients may refer to less proven treatments, which can lead to the delay of standard therapy [11]. In China, the online medical information is often shown side-by-side with commercial promotions, which undermines its independency and accuracy. The government should have strict laws against false advertising of online medical information and recommend credible information sources to the public. Physicians should help patients by referring them to reliable and patient-friendly websites and communicating with the patients online.

This study has some limitations. As a study undertaken in a single surgical unit, this population may not be typical of the Chinese population as a whole; in particular, we did not include patients with late-stage cancer or those who were receiving chemotherapy. In addition, we did not analyze some factors that may influence the DFS results, such as time duration from breast tumor detection to initial treatment, adjuvant therapy (radiation therapy, chemotherapy, and targeted therapy), complications of surgery and adjuvant therapy, compliance to therapy, and web-searching behavior after surgery. These factors should be included in future studies. In addition, a multicenter study with larger patient numbers would increase the sample size and add more weight to these results.

Findings from this study have significant implications for clinical practice. First, physicians should ask patients with breast cancer about their use of the internet as an information source and help them to identify inaccurate and misleading information. Second, physicians should discuss the online information with their patients and help them to better understand the disease and treatment options. Third, health care providers should direct patients to accurate and credible websites and provide high-quality online medical information. When discussions of online medical information between physicians and patients are incorporated into the consultations, both shared clinical decision making and the physician-patient relationship will be improved [18].

In conclusion, patients with breast cancer frequently use the internet to obtain health-related information. This study has shown that patients who are most likely to use the internet for breast cancer information are younger and well-educated, and they are more likely to have BCT. Web-using patients who were concurrently satisfied with internet information showed significantly improved DFS compared to those who did not search online for medical information. Patients should browse credible websites offering accurate and updated information, and website developers should provide high-quality and easy-to-understand information to better meet the needs of patients with breast cancer.

## Abbreviations

BCT: breast conserving therapy
DFS: disease-free survival
OR: odds ratio
PUMCH: Peking Union Medical College Hospital.
